# Glycogen is a neutral cargo of bulk autophagy in *Komagataella phaffii*

**DOI:** 10.1080/27694127.2025.2467454

**Published:** 2025-02-19

**Authors:** Nimna V. Wijewantha, Taras Y. Nazarko

**Affiliations:** Department of Biology, Georgia State University, Atlanta, USA

**Keywords:** CBM20, glycogen granules, glycogen synthase, glycophagy, yeast

## Abstract

Glycogen is a primary cellular energy store in numerous eukaryotes. Its biosynthesis is a main strategy to cope with forthcoming starvation. During starvation, glycogen is processed in the cytosol or delivered for degradation to animal lysosomes or yeast vacuoles by macroautophagy (hereafter autophagy). However, the mechanism of glycogen autophagy is poorly understood, especially in the heart and skeletal muscles that suffer from the lysosomal glycogen accumulation in Pompe disease. We recently developed the *Komagataella phaffii* yeast as a simple model to study glycogen autophagy and found that this pathway proceeds non-selectively. However, studies in *Saccharomyces cerevisiae* proposed glycogen as a non-preferred cargo of bulk autophagy. In our latest study with new fluorescent reporters for glycogen, we clarified cargo properties of *K. phaffii* glycogen. Both homologous and heterologous markers of glycogen are delivered to the vacuole and degraded with efficiencies that are independent of glycogen, suggesting that glycogen is a neutral cargo of bulk autophagy. This work provides insights into the evolutionary diversity of glycogen autophagy in yeasts with implications for understanding this process in complex eukaryotes.

Glycogen is the primary storage form of glucose in many eukaryotic organisms. Its biosynthesis begins with glycogenin, which initiates the process by attaching the first glucose molecule to itself and then by elongating it with additional glucose units via α-1,4-glycosidic bonds. The glycogen synthase quickly takes over the elongation process, while a branching enzyme introduces α-1,6-glycosidic bonds, forming the highly branched structure of glycogen. This structure becomes the core of glycogen granules, which also contain several other proteins involved in glycogen metabolism.

During starvation, cells utilize glycogen through phosphorolysis and hydrolysis. The latter requires the delivery of glycogen from the cytosol to animal lysosomes or yeast vacuoles by macroautophagy, hereafter autophagy. Autophagy is a transport pathway that can sequester intracellular components within autophagosomes and deliver them to lysosomes/vacuoles. While autophagy can be selective by targeting specific organelles or even macromolecules, it can also proceed in a non-selective, bulk manner when autophagosome precursor, the phagophore, indiscriminately encloses cytoplasmic material.

In mammals, the accumulation of glycogen in lysosomes due to mutations in the gene of the lysosomal acid α-glucosidase leads to the Pompe disease, which is characterized by a severe heart and skeletal muscles dysfunction. While autophagy is a primary mechanism for the lysosomal delivery of glycogen, whether autophagy is selective or non-selective remains unclear, particularly in the heart and skeletal muscles where glycogen autophagy is independent of the autophagic receptor for glycogen, STBD1 (starch binding domain 1).

Previously, we developed *K. phaffii* yeast as a simple model for glycogen autophagy. Using the GFP-tagged glycogenin, *Kp*Glg1-GFP, as a marker for glycogen, we determined that it is degraded by bulk autophagy under nitrogen starvation conditions. However, a recent study in *S. cerevisiae* suggested that glycogen is a non-preferred cargo of bulk autophagy with only a limited sequestration into autophagosomes through the selective autophagy receptor, *Sc*Atg45.

In our latest study [1], we explored the cargo properties of *K. phaffii* glycogen using new fluorescent reporters. First, we employed the GFP-tagged glycogen synthase (*Kp*Gsy1-GFP) to monitor glycogen autophagy in *K. phaffii*, similarly to how it was done in *S. cerevisiae* using *Sc*Gsy2-GFP. Since *K. phaffii* has only one glycogen synthase, *Kp*Gsy1, this protein is the only ortholog of the *Sc*Gsy1 and *Sc*Gsy2 paralogs. First, we proved that *Kp*Gsy1 is indeed associated with glycogen granules by confocal microscopy. Then, we showed that *Kp*Gsy1-GFP can be exploited to monitor glycogen autophagy since *Kp*Gsy1-GFP was delivered into the vacuole and degraded in the autophagy-dependent manner, as we previously established for *Kp*Glg1-GFP. Therefore, *Kp*Gsy1-GFP can indeed be used as an alternative reporter to monitor this process.

*S. cerevisiae* glycogen is largely spared from degradation during the first 24 hours of nitrogen starvation, as evidenced by very limited processing of *Sc*Gsy2-GFP reporter in cells containing glycogen (when it reports about glycogen autophagy) and much better processing of the same reporter in cells lacking glycogen (when it reports about the autophagy of cytosol). In contrast, the *Kp*Gsy1-GFP reporter does not show such an effect in *K. phaffii* [1]. Experiments in glycogen-proficient and -deficient cells showed that glycogen affects neither vacuolar delivery nor degradation of *Kp*Gsy1-GFP. These findings suggest that glycogen is a neutral cargo of bulk autophagy in *K. phaffii*.

We verified our findings by developing two new glycogen reporters based on the glycogen binding domain – carbohydrate-binding module, family 20 (CBM20) – from the human STBD1 protein, GFP-CBM20 and mCherry-CBM20 [1]. Like *Kp*Glg1-GFP and *Kp*Gsy1-GFP, the GFP-CBM20 and mCherry-CBM20 reporters localized to glycogen granules in *K. phaffii* making them independent heterologous markers for these structures. Our experiments revealed that the vacuolar delivery and degradation of CBM20 fusions depend on various components of autophagy machinery, confirming their utility as reporters to monitor glycogen autophagy. Like *Kp*Gsy1-GFP, the CBM20-based reporters exhibited essentially the same vacuolar trafficking and processing patterns in glycogen-proficient and -deficient cells. This result confirmed that glycogen behaves as an inert cargo of bulk autophagy in *K. phaffii*.

The studies in *K. phaffii* and *S. cerevisiae* reveal fundamental species-specific differences in glycogen turnover by autophagy during nitrogen starvation (Figure 1). In *K. phaffii*, glycogen granules are neutral substrates of bulk autophagy, as most cytosolic proteins are [1], whereas in *S. cerevisiae*, they are initially excluded from the sequestration within autophagosomes until they are selectively targeted by *Sc*Atg45, upon increased expression of this selective autophagy receptor due to prolonged starvation or sporulation. Yet, Atg45 may be restricted to *Saccharomycetaceae* family and *Candida albicans*, raising questions about the existence of such selective autophagy receptor and selective autophagy of glycogen (glycophagy) in other yeast species, including *K. phaffii*.

**Figure 1.**
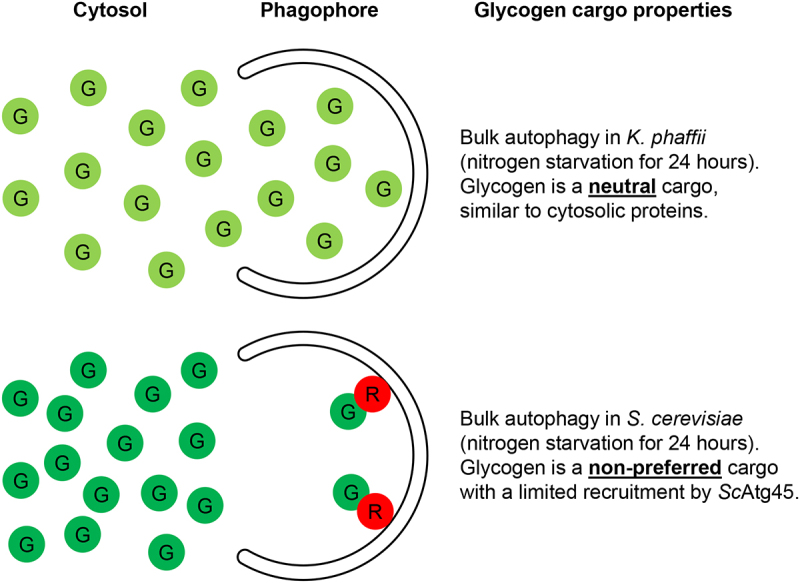
Differences in glycogen autophagy between *K. phaffii* and *S. cerevisiae*. While glycogen is a neutral cargo of bulk autophagy in *K. phaffii*, it is a non-preferred cargo of bulk autophagy in *S. cerevisiae* (which is partially compensated for in this yeast species by the selective autophagy receptor for glycogen *Sc*Atg45). G, glycogen granule; R, *Sc*Atg45.

These findings in yeast pave the way for future studies of glycogen autophagy in mammalian systems, particularly skeletal and cardiac muscles in which glycogen autophagy is independent of STBD1, the established glycophagy receptor in the liver. The STBD1 independence of glycogen autophagy in muscles raises an important question whether glycogen is a neutral or a selective cargo of autophagy in these tissues. Addressing this question is crucial to understand glycogen autophagy in non-hepatic tissues and has direct implications for Pompe disease, since if glycogen is a selective cargo, then a so far unknown glycophagy receptor in muscles will constitute a highly desirable target for the treatment of Pompe disease.

## Data Availability

No new data were created or analyzed in this study.

## Abbreviations

CBM20, carbohydrate-binding module, family 20; GFP, green fluorescent protein; STBD1, starch binding domain 1.
